# High temperature heat flux sensor with ITO/In_2_O_3_ thermopile for extreme environment sensing

**DOI:** 10.1038/s41378-024-00748-8

**Published:** 2024-07-25

**Authors:** Helei Dong, Meimei Lu, Weifeng Wang, Qiulin Tan

**Affiliations:** 1 School of Instrument and Electronics, North University of China, 030051 Taiyuan, China; 2 Key Laboratory of Micro/nano Devices and Systems, Ministry of Education, North University of China, 030051 Taiyuan, China

**Keywords:** Sensors, Structural properties, Electrical and electronic engineering

## Abstract

Hypersonic vehicles and aircraft engine blades face complex and harsh environments such as high heat flow density and high temperature, and they are generally narrow curved spaces, making it impossible to actually install them for testing. Thin-film heat flux sensors (HFSs) have the advantages of small size, fast response, and in-situ fabrication, but they are prone to reach thermal equilibrium and thus fail during testing. In our manuscript, an ITO–In_2_O_3_ thick film heat flux sensor (HFS) is designed, and a high-temperature heat flux test system is built to simulate the working condition of a blade subjected to heat flow impact. The simulation and test results show that the test performance of the thick-film HFS is improved by optimizing the structure and parameters. Under the condition of no water cooling, the designed HFS can realize short-time heat flux monitoring at 1450 °C and long-term stable monitoring at 1300 °C and below. With a maximum output thermopotential of 17.8 mV and an average test sensitivity of 0.035 mV/(kW/m^2^), the designed HFS has superior high-temperature resistance that cannot be achieved by other existing thin (thick) film HFSs. Therefore, the designed HFS has great potential for application in harsh environments such as aerospace, weaponry, and industrial metallurgy.

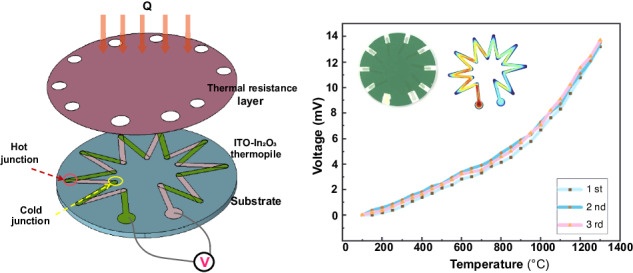

## Introduction

In recent years, the analysis of thermal phenomena has received increasing attention from research scholars. Heat flow density, as an important thermal parameter representing the heat transfer per unit area, plays an important role in production and life^1–3^.

Heat flow density is one of the key parameters to be measured in many applications, such as gas turbine engines, aero-engine turbine blade surfaces, and other high heat flux environments. Accurate heat flow measurements will help to better monitor and control the operating process of high-temperature systems, monitor equipment performance in real time and optimize thermal protection systems^4^.

There are many types of conventional heat flux sensors(HFSs), such as Gardon-type sensors^5–7^, Schmidt–Boelter sensors^8^, and gradient-type heat flow sensors^9–11^. However, the presence of traditional HFSs can bring large errors to the measurement environment due to their large size and complex installation, such as the introduction of flow field disturbance; in addition, they generally can only work at limited temperatures or heat flux densities and have a long response time^12^. Compared to the above types of HFSs, thin-film HFSs have attracted more research interest due to their small size, thin thickness, and fast response^13–15^, especially the thermopile-type thin-film HFSs, which have a great advantage in high-temperature harsh environments. In addition, in recent years, as the performance of aero-engines continues to improve, their operating temperatures also continue to increase, so the demand for high-temperature thin film HFSs has risen dramatically^16–18^.

Among all kinds of thin film HFSs, thermoelectric stack type thin film HFSs stand out due to their large output, high sensitivity, and fast response^19–21^. Researchers have carried out systematic and extensive research on thermoelectric stack type thin film HFSs, which are mainly composed of three parts: substrate, thermoelectric stack sensitive layer, and thermal resistance layer^12,22–28^. The substrates are mainly made of two materials, ceramic or metal, with ceramic substrate Al_2_O_3_^2,17,22,25,27,28^ predominating and, to a lesser extent, AlN ceramics^12,18^. The sensitive layer is mainly made of metals and metal oxides, most of which are platinum rhodium precious metals Pt-Pt/Rh^17,19,22,23,28^ and ITO–In_2_O_3_^2,21,24,27^, and some are other precious metals, such as constantan^25^ and tungsten rhenium thermocouples^15^. The main materials for the thermal resistance layer are alumina^2,19^, silicon dioxide^16,17,20–22,24,26^, YSZ^27^, etc.

Li et al.^2^ fabricated an ITO–In_2_O_3_ thermoelectric stack thin film heat flux meter using the magnetron sputtering method on a nickel alloy substrate, with a sensitivity of 61.93 μV/(kW/m^2^) and a maximum operating temperature of 888 °C. Li et al.^26^ fabricated a PDC thermoelectric stack thin film heat flux sensor (HFS) using the 3D printing method on an alumina ceramic substrate, with a sensitivity of 1.349 mV/(kW/m^2^) and a maximum operating temperature of 800 °C. Fu et al.^15^ prepared 136 pairs of W–26Re/W–5Re thermopile thin-film HFSs on aluminum nitride ceramic substrates by magnetron sputtering, the design added an alumina protective layer above the sensitive layer, and its maximum operating temperature was 1000 °C, and the maximum output thermopotential was 3 mV. Wang et al.^14^ designed and fabricated a new sandwich structure Pt/PtRh13 thermopile thin-film HFS, the sandwich structure can increase the temperature difference between the hot and cold junctions and thus improve the output, and the maximum operating temperature of this sensor was 1000 °C. Xie et al.^22^ prepared 12 pairs of Pt/Pt–10Rh thermopile thick-film HFSs on an alumina ceramic substrate with a tested average sensitivity of 0.0056 mV/(kW/m^2^), its maximum operating temperature of 1200 °C, and a maximum output voltage of 1.41 mV. However, most existing thin film HFSs have limited operating temperatures (generally not exceeding 1000 °C)^2,17,19–21,23,26^, and only a few thick film HFSs can break through higher temperatures^22,24^. Therefore, existing HFSs still face difficulties in high-temperature environments, such as surface applications in aircraft engines.

Precious metals have lower Seebeck coefficients and are prone to failure due to oxidation at high temperatures. Transparent semiconductor oxides (such as ITO, In_2_O_3_, etc.) are gradually showing their prominent advantages at high temperatures, such as high-temperature resistance, large thermal potential output, excellent electrical conductivity, high-temperature stability, oxidation resistance^29–33^, etc. These advantages allow us to see the possibility of semiconductor oxides as thermoelectric-sensitive materials for applications in high-temperature and harsh environments such as aerospace. Therefore, ITO and In_2_O_3_ are used as thermoelectric conversion materials to study the high-temperature HFS.

In this work, we designed and fabricated a novel structure of ITO–In_2_O_3_ thermopile-type thick-film HFS, which consists of an alumina ceramic substrate, an ITO–In_2_O_3_ thermopiles sensitive layer, and a thermal resistance layer with lower thermal conductivity. The thermal resistance layer of the present structure covers as much as possible of the sensitive layer underneath to prevent its sublimation at high temperatures. We carried out finite element simulations, observed the microscopic morphology with a scanning electron microscope, and built a static、dynamic test platform. With the dual effects of material selection and structural optimization, the proposed HFS has excellent temperature resistance up to 1450 °C shortly and can work stably for a long period of time at 1300 °C, which can be applied in the process of aero-engine ignition, operation and so on. In addition, the sensor has prominent thermal potential output and sensitivity of 17.8 mV and 0.035 mV/(kW/m^2^), respectively.

## Materials and methods

### Working principle

The working principle of the HFS can be jointly explained by the first law of thermodynamics, Fourier’s principle of one-dimensional heat conduction, and the Seebeck effect. The first law of thermodynamics studies the heat transfer process in terms of energy conservation, which can be simplified to the following Eq. (1) in one-dimensional solid heat transfer:

1 $$\frac{{{{d}}}^{2}T}{{{d}}{x}^{2}}=0$$

According to Fourier’s one-dimensional heat conduction law, when there is a heat flow through the heat-resistive layer, there is a temperature difference between the upper and lower surfaces, and the heat flux *Q* can be defined as

2 $$Q=-\lambda \frac{{{{d}}T}}{{{d}x}}$$

Combining Eqs. (1) and (2) with the boundary conditions, we can obtain the specific relationship between the heat flux density, temperature difference, thermal conductivity, and the thickness of the thermal resistive layer:

3 $$Q=-\lambda \frac{{T}_{1}-{T}_{2}}{\delta }=\lambda \frac{{T}_{2}-{T}_{1}}{\delta }$$

where *Q* is the heat flux density, *λ* is the thermal conductivity of the thermal resistance layer material, *T*_2_ and *T*_1_ are the temperatures of the hot and cold junctions, respectively, and *δ* is the thickness of the thermal resistance layer.

Thermopiles are used as the sensing element and operate in conjunction with a heater element for thermoelectric sensing^34^. According to the Seebeck effect^35^, when two different materials (conductors or semiconductors) come into contact with a temperature difference, a thermoelectric potential is generated in the circuit, and the output expression is

4 $$E=N\cdot \Delta T\cdot {S}_{{{{AB}}}}$$

where *N* is the number of thermocouples, Δ*T* represents the temperature difference between the hot and cold junctions, and *S*_AB_ denotes the Seebeck coefficient of thermocouples.

Combining Eqs. (3) and (4), the sensitivity *S* of the HFS is obtained as

5 $$S=\frac{E}{Q}=\frac{N\cdot {S}_{{{{AB}}}}\cdot \delta }{\lambda }$$

### Structure design

**Fig. 1:**
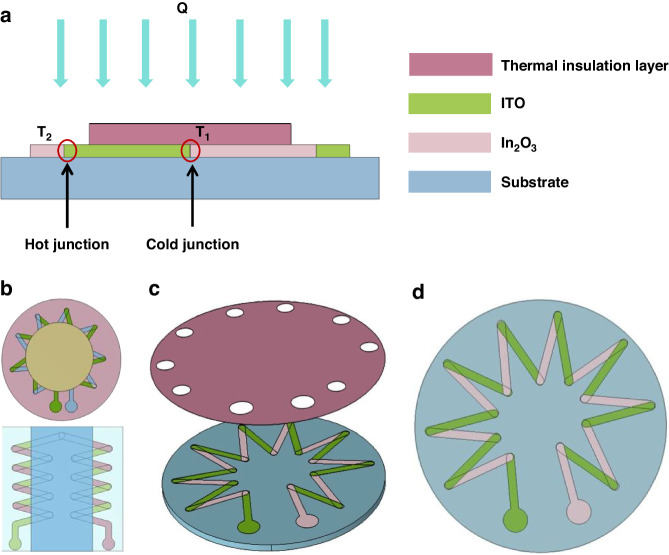
Structure diagram of HFS. **a** 2-D model. **b** Structure of universal thermopile type HFSs. **c** 3D structure diagram of designed HFS. **d** Schematic diagram of thermoelectric stack structure

The structure design is based on the working principle of the thin film HFS, which consists of three parts, including an alumina ceramic substrate, a thermoelectric stack sensitive layer connected by 9 pairs of ITO–In_2_O_3_ thermocouples, and a low thermal conductivity thermal insulation layer. As shown in Fig. 1a, when the heat flux *Q* is applied to the surface of the sensor, the low thermal conductivity of the thermal resistance layer prevents heat from transferring from the surface of the thermal resistance layer to the sensitive layer downwards. Therefore, below the thermal resistance layer is the cold junction with a temperature of *T*_1_, and the part not covered by the thermal resistance layer is the hot junction with a temperature of *T*_2_. This design aims to convert the temperature difference between the hot and cold junctions of the sensitive layer into an output thermoelectric potential for detection.

The general structure of the existing thermopile-type HFSs is shown in Fig. 1b, and the substrates are commonly round and square, and the arrangement of the thermopiles is adjusted with the shape of the substrates, and then a round or rectangular thermal resistance layer is covered above the sensitive layer of the thermopile. In this work, we choose a round substrate that can arrange more pairs of thermocouples, and unlike the previous ones, the thermal resistance layer of this design only exposes the hot junction, which can increase the temperature difference between hot and cold junctions, and at the same time better prevent the sensitive layer from being exposed at high temperature. Compared with most of the schemes that only cover the cold junction, the thermal resistance layer of this design covers as much sensitivity layer area as possible, which can increase the temperature difference between the hot and cold junctions, and at the same time, it can better prevent the sensitive layer of the thermopile from oxidizing at high temperatures. Figure 1c and d show the three-dimensional schematic of the designed HFS and the surface of the thermopile, respectively.

### Simulation

In order to verify the rationality of the designed HFS, and also to study the effects of the thickness of the thermal resistance layer and the input heat flux density on the temperature difference between the hot and cold junctions and the response time. The simulation parameters are shown in Table 1.

**Table 1:** Geometric parameters of the FEM model

Component	Geometric parameters	Value (μm)
Al_2_O_3_ ceramic substrate	Radius (*R*_0_)	15,000
	Thickness (*H*_0_)	1000
ITO–In_2_O_3_ thermocouple strip	Length (*L*_1_)	8000
	Width (*W*_1_)	1000
	Thickness (*H*_1_)	20
Thermal insulation layer	Radius (*R*_2_)	15,000
	Thickness (*H*_2_)	35

**Fig. 2:**
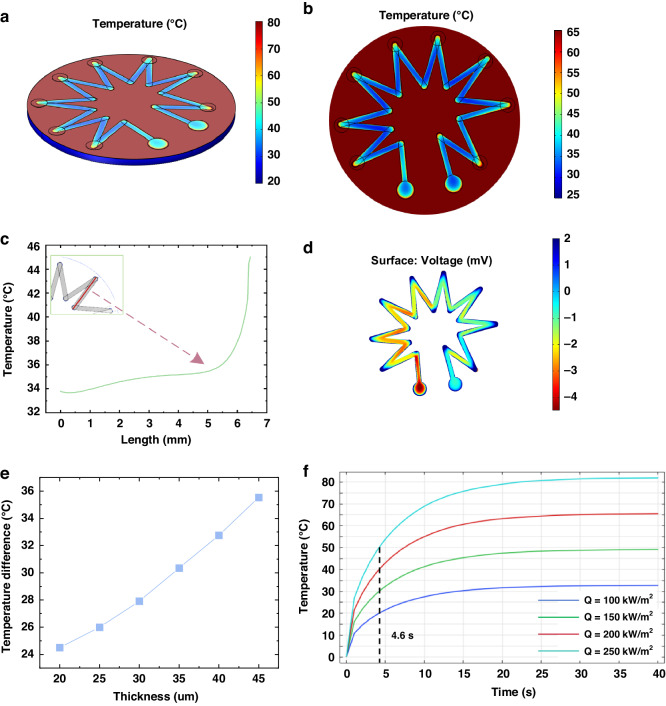
Simulation results of the designed HFS. **a** 3D-temperature distribution. **b** Temperature distribution on thermopile layer. **c** Thermocouple temperature distribution line diagram. **d** Electric potential distribution on thermopile layer. **e** Variation of temperature difference with thickness of thermal resistance layer. **f** Transient simulation results with different heat flux densities

A heat flux of 200 kW/m^2^ is vertically applied to the upper surface of the HFS. The primary temperature was set at 293.15 K, and the back of the alumina substrate was set to a constant temperature condition of 293.15 K. The convective heat transfer coefficient on the sides of the substrate and thermal resistance layer is set to 10 kW/m^2^. One end of the thermopile is set to ground, and the other end is set to floating voltage. The temperature distribution and potential distribution are shown in the Fig. 2.

As shown in Fig. 2a and b respectively show the overall temperature distribution and the temperature distribution of the surface of the thermopile, and Fig. 2c shows the temperature distribution line map of one of the thermocouples. It can be seen that the average temperature at the hot junction of the sensor is higher than the average temperature at the cold junction when it reaches a steady state. Figure 2d shows the cross-sectional potential distribution of the thermoelectric stack when the heat flux density is 200 kW/m^2^. It can be seen that the temperature difference between the hot and cold junctions and the thermopotential output are significant.

The thickness of the thermal resistance layer has a significant impact on the temperature difference between the hot and cold junctions and the output thermoelectric potential. In order to explore the optimal thickness of the thermal resistance layer, a parameter scan of the thermal resistance layer thickness was added, and the thickness was set to six values: 20, 25, 30, 35, 40, and 45 μm. The effect of the thermal resistance layer thickness on the temperature difference between the hot and cold junctions was observed in the results, as shown in Fig. 2e It can be seen that as the thickness of the thermal resistance layer increases, the temperature difference between the hot and cold junctions increases linearly. When the thickness of the thermal resistance layer is 20 μm, the temperature difference is 24.6 °C. When the thickness increases to 45 μm, the temperature difference between the hot and cold junctions increases to 35.6 °C. From this, it can be seen that within a certain range, the thicker the thermal resistance layer, the better. But as the thickness increases, the response time of the sensor also increases, and the response slows down. In summary, the selection of the thickness of the thermal resistance layer should be comprehensively considered, and the thickness of the thermal resistance layer selected in this design is 35 μm.

Figure 2f shows the transient simulation results of the designed HFS for different heat flux densities. A step heat flux is applied to the upper surface, and the time constant of the first-order response is defined as the difference between the moments corresponding to the increase of the output from the moment of 0–63.2% of the steady-state value and the time constant of the transient simulation is calculated to be 4.6 s. From the figure, it can be seen that the temperature difference between the hot and cold junctions rises with the increase of applied heat flux, and at the same time, we find that the change of heat flux densities has no effect on the time-constant, which suggests that input heat flows do not affect the dynamic characteristics of the sensor.

## Fabrication, characterization and package fabrication

### Fabrication

**Fig. 3:**
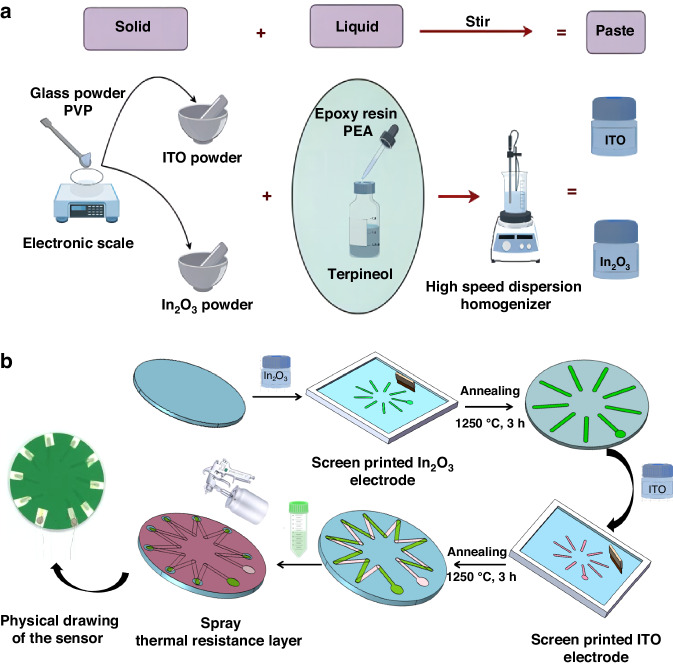
Fabrication process of the HFS. **a** ITO, In_2_O_3_ paste preparation. **b** Sensor preparation process

Figure 3 shows the preparation process of the HFS, screen printing is used to prepare the ITO and In_2_O_3_ sensitive layer, and the thermal insulation layer is fabricated by spraying method combined with mask. The whole preparation process needs to be combined with heat-treated technology to eliminate internal defects in the material and improve performance.

First of all, we configure the ITO and In_2_O_3_ pastes, and we need to configure the corresponding solid phase and organic solvent first. The required raw materials include ITO powder, In_2_O_3_ powder (Nangong Fenghui Nano Technology Co.), binder—glass powder, dispersant—polyvinyl pyrrolidone (PVP), solvent—Terpineol, thickener—Epoxy resin, cosolvent—polyetheramine (PEA) (Shanghai Aladdin Biochemical Technology Co.). First, In_2_O_3_/ITO powder: binder: dispersant (PVP) = 65:8:2 is prepared into a solid phase, and then a certain proportion (solvent:thickener:cosolvent = 3:2:1) is configured into an organic solvent, and then solid-liquid mixing (solid content of 75%), using high-speed dispersing homogenizer (FJ300-SH) to fully mixing and stirring, made of ITO, In_2_O_3_ slurry. The preparation process is shown in Fig. 3a.

#### Substrate pretreatment

The substrate is made of 99 alumina ceramic, which is ultrasonically cleaned sequentially with acetone, ethanol, and deionized water and blown dry with a nitrogen gas gun for spare parts.

#### Screen printing preparation of sensitive layer

The ceramic sheet is attached to the front side of the screen-printed plate and then coated with an In_2_O_3_ paste on the other side. A soft squeegee was used to apply the paste under pressure, and the ceramic sheet was removed for drying (125 °C for 15 min). The ITO electrodes were then formed in the same way and annealed in a chamber muffle furnace at a heating rate of 5 °C/min to 1250 °C for 3 h. In this way, the preparation of ITO–In_2_O_3_ thermoelectric sensitive layer was completed.

#### Preparation of thermal resistance layer

The purchased material is nano-composite ceramic (YS-8809 of China Yousheng New Material Company), which is composed of curing agent A, alumina, and silicon oxide nano-composite ceramic B. The two components were weighed and mixed together in a ratio of 1:1 and shaken for about 20–30 min until the coating was fully matured. Then start the spraying cycle until the desired thickness of 35 μm is achieved.

#### Lead preparation

After the thermal resistance layer is completely dried, a platinum wire is bonded to the connection of the two leads with platinum paste. Then drying and high-temperature sintering complete the preparation of the leads.

### Characterization

**Fig. 4:**
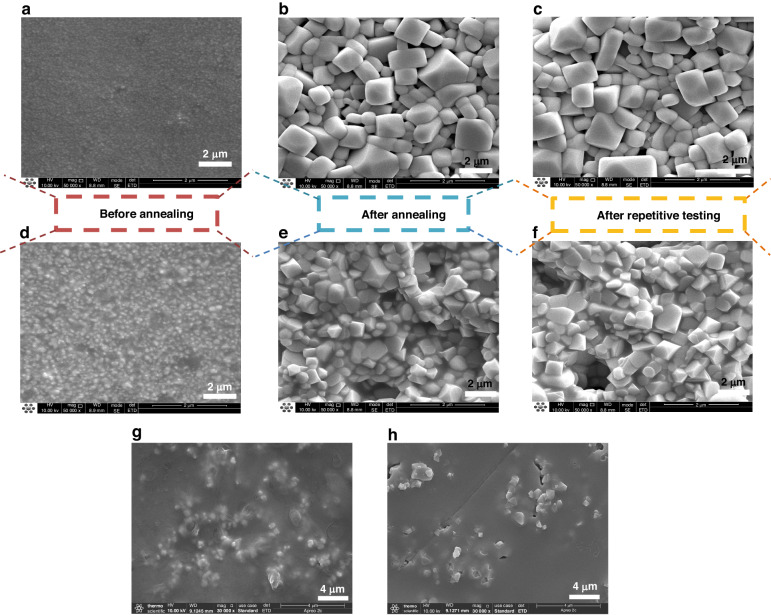
The SEM images of the HFS under different thermal environments. The SEM images of In_2_O_3_ electrodes **a** before annealing, **b** after annealing, and **c** after repetitive testing. The SEM images of ITO electrodes **d** before annealing, **e** after annealing and **f** after repetitive testing. The SEM image of the thermal resistance layer after **g** spraying and **h** high-temperature testing

In order to further understand the properties of the thermal electrodes and thermal resistance layer materials and their microscopic morphology, we characterized the microstructures of the ITO and In_2_O_3_ thermal electrodes as well as the thermal resistive layer at different stages by using a scanning electron microscope SEM (FEI INSPECT F50, USA). As shown in Fig. 4, we characterized the microstructures of ITO and In_2_O_3_ electrodes before annealing, after annealing at 1250 °C, and after repeatability testing at 1300 °C, respectively. As can be observed from Fig. 4a and d, the surface of the unannealed In_2_O_3_, ITO electrodes is flat, dense, well-connected, and free of obvious cracks and holes, which is sufficient to ensure the good thermoelectric properties of the thermocouple films.

Figure 4b and e show the microscopic images of In_2_O_3_ and ITO after annealing at 1250 °C, respectively. It can be seen that the grain size increases significantly after annealing, and the organic carriers (epoxy resin, Terpineol, PEA) in In_2_O_3_ completely volatilize after annealing at 1250 °C, resulting in dense cubic crystal particles on the surface. The surface of ITO is not flat, which may be due to the fact that the Sn in ITO volatilizes at high temperatures, resulting in the imbalance of the interlayer stress.

Figure 4c and f show the SEM microscopic images of In_2_O_3_ and ITO after testing at a high temperature of 1300 °C. It can be seen that the difference between the images after testing and annealing is tiny and almost unchanged, which indicates that thermopile can withstand high temperature testing.

Figure 4g and h show the SEM images of the thermoresistive layer after spraying and after high-temperature testing, respectively. From the figures, it can be seen that the thermal resistance layer prepared by the spraying process presents nearly spherical particles, and this morphology can increase the mobility of the powder during the spraying process and improve the deposition efficiency of the coating. After the high-temperature test, the particles have a tendency to increase in size, and a few small cracks grow in the thermal resistive layer due to thermal stress, but this does not affect the thermal resistive properties of the thermal resistive layer.

### Package

**Fig. 5:**
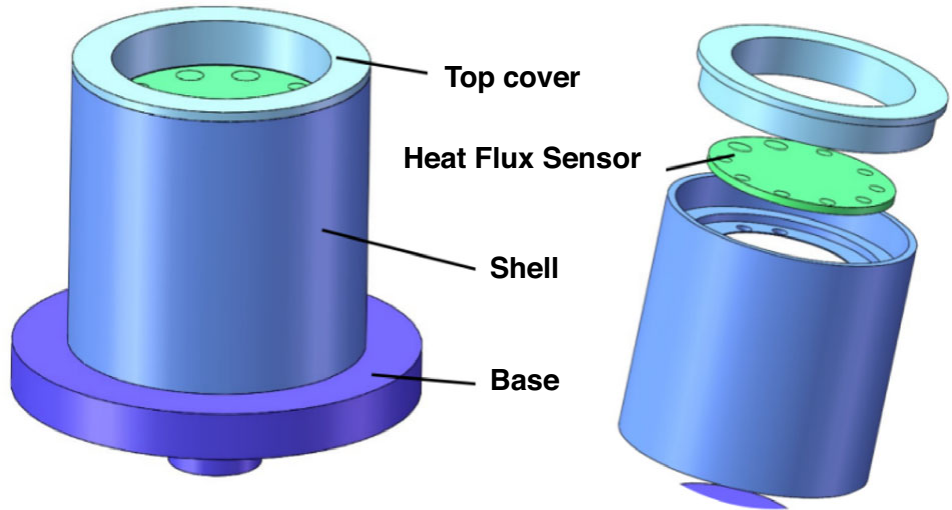
Schematic diagram of the structure. Heat flux sensor package design and assembly

Thermal equilibrium is the most common problem in the testing of HFSs. When the heat rises to a certain temperature and lasts for a certain period of time, the accumulated heat in the sensitive layer begins to change from longitudinal to transverse heat transfer so that the temperature of the hot and cold junctions converge, and thus there is no thermopotential output. Therefore, we add a ceramic package to draw out the heat from the back of the HFS in time. The structure of the package is shown in Fig. 5, which consists of a top cover, a shell, and a base. The HFS is put into the round hole reserved in the shell, and the top cover fixes the sensor in the shell. The inside of the shell is provided with lead holes for leading out the signal of the HFS. When the front side of the sensor is heated, the thermal resistance layer makes the temperature difference between the hot and cold junctions, and the heat at the back of the base is led out in time through the ceramic, which effectively prevents the occurrence of lateral heat transfer and keeps the temperature at the cold end basically constant, so as to enable the HFS to have a normal potential output.

## Experimental results and discussion

**Fig. 6:**
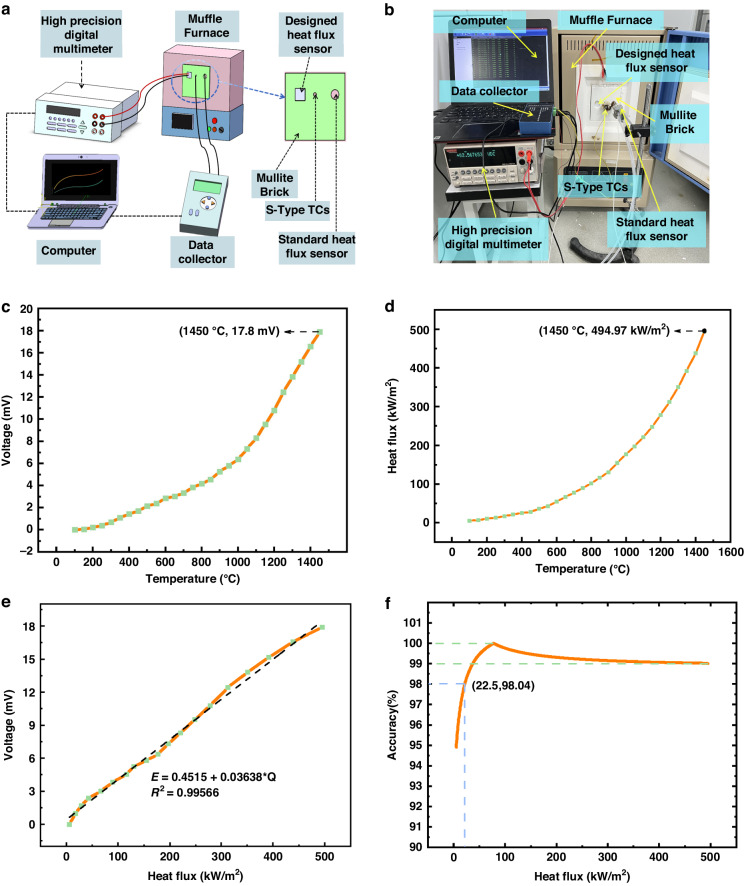
The test platform of HFS and extreme testing results. **a** Schematic diagram. **b** Physical test diagram. **c** Voltage–temperature curve of the designed HFS. **d** Heat flux density–temperature curve of the standard HFS. **e** Voltage–heat flux density curve of the designed HFS. **f** The accuracy of the designed HFS

In order to better understand the static-dynamic performance of the designed HFS, we built a static-dynamic calibration platform for HFS. The static test platform is shown in Fig. 6, and the test instruments consist of a muffle furnace (KSL-1700X, China), the designed HFS, a GD standard heat flow meter, a standard S-type thermocouple, a high-precision digital multimeter (KE2002, China), a data acquisition card and a computer.

### Static testing

The static test adopts the comparative calibration method. Standard S-type thermocouple is used to calibrate the temperature inside the furnace, the standard heat flow meter is used to calibrate the output voltage of the designed HFS, and a high-precision digital multimeter and data acquisition card will transmit the collected signal to the computer for display.

As shown in Fig. 6a and b, the temperature of the muffle furnace is set to increase from room temperature to 1450 °C. It can be seen from the figure that both the output thermopotential and the heat flow density increase with the temperature, the maximum output voltage is 17.8 mV, and the maximum heat flow density is 494.97 kW/m^2^ when the temperature rises to 1450 °C. It can be shown that the designed HFS has the ability to survive in very high-temperature environments.

From Fig. 6c–e can be made, and combined with the theoretical Eq. (5), it can be seen that the slope of the voltage-heat flow density curve is the sensitivity of the designed HFS. The linear fitting results are as follows:

6 $$\begin{array}{c}E=\,0.4515\,+0.03638\times Q\\{R}^{2}=0.99566\end{array}$$

In order to reveal the maximum deviation between the measured and the desired value, the accuracy of the sensor was calculated on the basis of a linear fit. In this example, based on the measured voltage *Y*_m_ and the fitted voltage *Y*_f_, the accuracy *δ*_Y_ can be defined as

7 $${\delta }_{{{Y}}}=\left(1-\left|\frac{{Y}_{{{m}}}-{Y}_{{{f}}}}{{Y}_{{{f}}}}\right|\right)\times 100 \%$$

The accuracy under different heat flows is shown in Fig. 6f. It can be seen that at the beginning of the operation, the muffle furnace power is unstable and the initial heating in the chamber is not uniform, which leads to a large difference between the fitted voltage and the measured voltage, and the calculated accuracy is low at 94.9%, and after the heat flow density is >22 kW/m^2^, the thermal environment is gradually stabilized, and the accuracy stabilizes at more than 98%, so it can be assumed that the accuracy of the designed HFS is 98%.

**Fig. 7:**
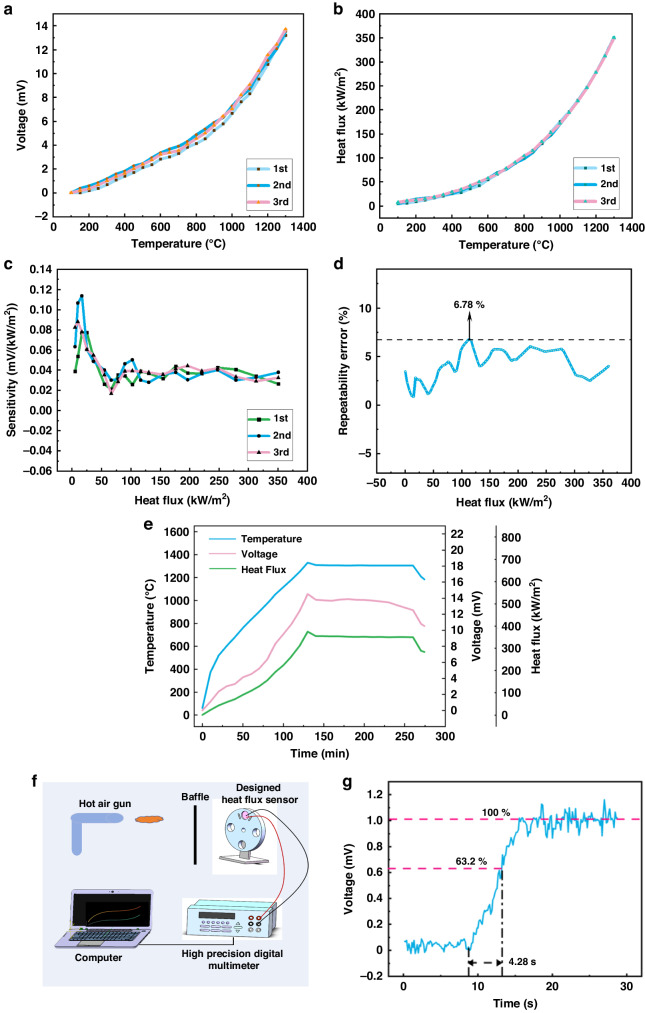
Repeatability testing, durability testing, and dynamic testing of HFSs. **a** and **b** Designed and standard HFS repeatability curves. **c** Sensitivity distribution curve of the designed HFS. **d** Percentage error of repeatability testing. **e** High-temperature holding experiment. **f** Dynamic test platform. **g** Dynamic response results

Then, we performed three repeatability tests, and the results are shown in Fig. 7a and b. When the temperature was increased from room temperature to 1300 °C, the maximum output potential and the maximum heat flow density at the highest temperature of 1300 °C are shown in Table 2. Analyzing the data yields a curve of sensitivity versus heat flow density, as in Fig. 7c. As can be seen from the figure, there are large voltage changes, and sensitivity jumps at the beginning of the operation, and the sensitivity tends to stabilize when the heat flow density is larger than 35 kW/m^2^, and the average sensitivity is 35.77 μV/(kW/m^2^).

**Table 2:** Repeatability test limit data

Times	Temperature (°C)	Voltage (mV)	Heat flux (kW/m^2^)
1st	1300	13.81	350.49
2nd	1300	13.54	351.85
3rd	1300	13.71	350.84

The repeatability error calculated from the following Eq. (8) is plotted in Fig. 7d:

8 $${\delta }_{{{R}}}=\frac{{|}\varDelta {R}_{max }{{|}}}{{Y}_{{{{FS}}}}}\times 100 \%$$

where *Y*_FS_ is the full-scale value of the system and $\Delta {R}_{\max }$ is the maximum difference between the outputs of the same input corresponding to multiple cycles of the same stroke. From Fig. 7d, we can see that the percent error remained relatively stable during the test, with a maximum percent error of 6.78%. It can be concluded that the designed HFS has good consistency.

Finally, in order to further investigate the high-temperature performance of the prepared HFS, we performed high-temperature endurance tests. Figure 7e depicts the output of the designed HFS after heating up to 1300 °C and continuing for 2 h. It can be seen that during the high temperature holding phase, the temperature remains constant, and the heat flow and output potential show a slight decreasing trend. The heat flow was 355.52 kW/m^2^ at the beginning of the high temperature holding state and decreased to 350.49 kW/m^2^ after 2 h, with a reduction error of 1.41%. This phenomenon can be explained by the fact that as the hold time increases, some of the heat from the hot end is transferred to the substrate, resulting in a decrease in the temperature difference between the hot and cold junctions and a decrease in output.

A comparison of several sensors is given in Table 3, which shows that most of the current thin-film HFSs are not able to operate above 1200 °C. The sensor proposed in this study shows a significant improvement in the operating temperature. At the same time, it can be seen that due to the large Seebeck coefficients of ITO and In_2_O_3_, the increase in temperature resistance does not sacrifice the sensitivity of the sensor, which is still high.

**Table 3:** Comparison of different HFSs

Number	Materials	Tmax (°C)	Preparation process	Voltage (mV)	Sensitivity mV/(kW/m^2^)	Response time	Reference
1	ITO–In_2_O_3_	888	Sputtering	16	0.062	No	^2^
2	Pt/Pt–13Rh	1000	Sputtering	1.1	0.0115	15.24 ms	^17^
3	W–5Re W–26Re	1000	Sputtering	4	0.0038	No	^18^
4	Pt/PtRh10	1200	Screen printing	1.41	0.0056	No	^22^
5	PSN_2_/TiB_2_	800	3D-printing	250	1.349	0.9 s	^26^
6	Si/Au	1200	DRIE	60	0.0059	8 ms	^30^
7	ITO/In_2_O_3_	1450	Screen printing	17.8	0.035	4.28 s	This study

### Dynamic testing

In order to characterize the dynamic properties of the designed HFS, we built a dynamic test platform, as shown in Fig. 7f, which mainly consists of a hot air gun, a baffle plate, the designed HFS, a clamping device, a high-precision multimeter, and a computer. We fixed the HFS to be tested on the circular clamping device, at the same time, the HFS lead was connected to a high-precision multimeter to collect the output voltage, the multimeter was connected to a computer to display the real-time voltage data, and the heat gun was placed directly in front of the designed HFS to provide a heat flow source. Turning on the heat gun and applying a step heat flow source, the response of this first-order system is approximately an exponential curve, as shown in Fig. 7g.

The time constant is an important parameter that reflects the dynamic characteristics of a first-order system. According to the definition of the time constant in the previous text, we can obtain from Fig. 7g that the time constant of the designed HFS is 4.28 s, and when the output reaches a steady state, the output voltage is ~1.04 mV.

From Table 3, it can be seen that although the increase in thickness improves the temperature resistance, it will sacrifice the response time to some extent. In addition, in this paper, in order to be closer to the aerospace conditions, the hot air gun is chosen as the transient excitation heat source, compared with the use of laser as the excitation heat source in the literature^2,26,30^, whose heat transfer is supposed to be a slow process, so it makes the response time of the sensor larger. However, the response time is still substantial compared to conventional non-membrane heat flux sensors.

## Conclusion

In this paper, a novel thermopile-type HFS has been designed and fabricated, and the HFS can detect heat flow density at high temperatures up to 1450 °C. ITO and In_2_O_3_, which have a high melting point and are not easily oxidized, were chosen as the sensitive layer of the thermopile, and nanocomposite ceramics, which have a high melting point and lower thermal conductivity, were chosen as the thermal resistive layer. In addition, we have optimized the structure of the thermal resistance layer so that the area of the thermopile layer exposed to high temperatures is reduced, so the sublimation of ITO and In_2_O_3_ at high temperatures for a long period of time is prevented as much as possible, which greatly improves the temperature resistance of the sensor. The static-dynamic performance of the sensor was evaluated in a high-temperature environment, and the test results showed that the HFS can perform short detection at ultra-high temperatures up to 1450 °C and long-term operation at temperatures of 1300 °C and below, with a maximum output voltage of 17.8 mV and an average sensitivity of 0.035 mV/(kW/m^2^). Repeatability and durability tests have shown that the sensor can be stabilized at high temperatures for long periods of time. Therefore, the designed sensor can be used in high-temperature harsh environments such as aerospace.
